# Application of botulinum toxin to reduce the saliva in patients with amyotrophic lateral sclerosis

**DOI:** 10.1016/S1808-8694(15)31258-1

**Published:** 2015-10-20

**Authors:** Dayse Manrique

**Affiliations:** 1 Ph.D. in Medicine, Professor Department of Otorhinolaryngology, UNIFESP. AACD - Associação de Assistência à Criança Deficiente.

**Keywords:** salivary glands, botox®, sialorrhea, dysphagia, neuropathy, ALS

## Abstract

**Aim**: To demonstrate the effect of local application of Botox® in patients with amyotrophic lateral sclerosis (ALS), following our 2002 institutional protocol of sialorrhea treatment. **Study Design**: Clinical prospective. **Material and Method**: Five patients with ALS assisted at Clinic of Otolaryngology of AACD (Associação de Assistência à Criança Deficiente). They were all submitted to local application of Botox® in salivary glands and followed up for a year. The protocol consisted of clinical questionnaire about the inability of swallowing saliva and its repercussions in quality of life. Patients were submitted to previous odontological treatment, had intolerance to the adverse effects of anti-cholinergic agents and had not used Botox® for at least six months. The application was guided by ultrasound and the doses were 30U in one point for submandibular gland, and 20U in two points for each parotid gland, after topic anesthetic with prilocaine. **Results**: Five patients with ALS with sialorrhea, aged 45 to 59 years, were submitted to Botox® salivary glands application. We observed that the symptoms of sialorrhea changed dramatically in four patients. Three patients stayed almost four months without complaints with repercussion in quality of life. No patient presented local or systemic effects with local injection of Botox®.

## INTRODUCTION

The first report of saliva reduction with the use of toxin botulinum was in cats by Dickson and Shevry in 1923^1^. The use of botulinum toxin in salivary glands in vivo was reported primary in patients with amyotrophic lateral sclerosis (ALS) to block the action on cholinergic autonomic fibers ^1^.

In many neurological diseases, saliva stasis in the oral cavity and oropharynx and/or extraoral leak of saliva indicate neurogenic failure in coordination of tongue, palate and facial muscles that act in the first stage of swallowing.

About 50% of the patients with ALS have significant disorders in the control of saliva ^2^. Moreover, many other hundreds of patients with neurological diseases present this affection. These complaints collaborate to the social stigma of the disease, leading to difficult social integration, stressing depression and rehabilitation difficulties.

Among the treatment option, we can use drugs with anti-cholinergic effects, anti-Parkinson drugs, surgical treatment of salivary ducts or glands, radiotherapy in salivary glands, and more recently, application of botulinum toxin type A (Botox®) in salivary glands. Many patients present intolerance to adverse events of the used drugs, or they present very advanced and severe clinical conditions of the neurological disease preventing surgical treatment, and Botox® is the best alternative for treatment of sialorrhea. However, in the literature, population samples are small and heterogeneous, and doses, sites and application techniques are variable.

## OBJECTIVE

To demonstrate the results of the application of Botox®, in a prospective study in a homogenous population group, with standardized doses and techniques to reduce saliva and symptoms resulting from inability to control it, as well as the repercussions in quality of life.

## MATERIAL AND METHOD

In the period between December 2002 and December 2004, five patients with ALS with diagnosis for 2.8 years were consecutively selected in the Clinical Otorhinolaryngology area of AACD (Associação de Assistência à Criança Deficiente) for application of Botox® in the salivary glands. The follow up was performed on the 10th day after application (first application), and subsequent assessments were monthly for 12 months. The criteria used for inclusion were:

1. Diagnosis of ALS for at least 2 years;
2. Clinical manifestations suggestive of sialorrhea: accumulation of saliva in the oral cavity with continuous need for elimination, oral leak of saliva, difficulty to speak aggravated by accumulation of saliva in the oral cavity and pharynx;
3. Intolerance in the use of anti-cholinergic drugs;
4. No use of botulinum toxin in other sites for the past 6 months;
5. Dental and periodontal treatment before the application of Botox.

The questionnaire of results and quality of life was used on the application day (pre-application) and on each subsequent visit (post-application) and the responses were given in three categories (never, occasionally, frequently). The four questions were: need to eliminate saliva from the mouth, participation in the family group during the meals, embarrassment in public places because of sialorrhea, physical contact on the face with family and close friends. Treatment success was considered for the application and quality of life when at least three responses that used to be frequently before the application changed to never after the application.

### Application techniques

We performed topical anesthesia with prilocaine in the parotid gland (PG) and submandibular gland (SG) region thirty minutes before the application. The application of Botox® in the PG region was made in two points: 10 U in the angle between the posterior mandible angle and mastoid process and 10U in the angle between the zigomatic process and the ascending mandible process. In SG, we used ultrasound-guided application and injected 30 U in each gland in a central point.

**Figure 1:**
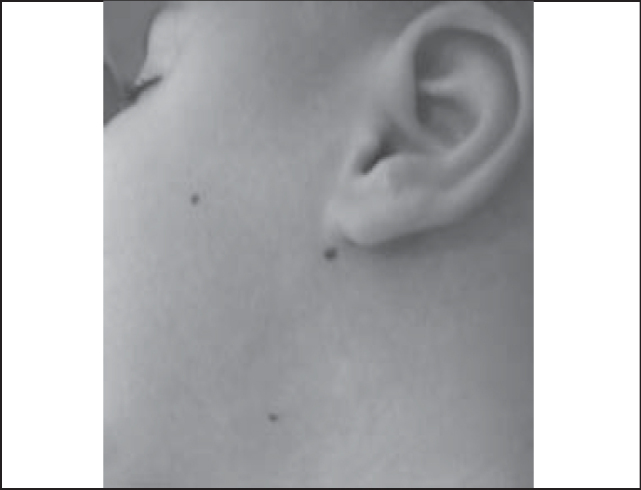
Application of toxin botulinum in salivary glands - Points of application of parotid gland (two points) and submandibular gland (one point).

## RESULTS

In the period between December 2002 and December 2004, five consecutive patients with ALS, aged between 45 and 59 years, three female and two male, were submitted to applications of Botox® to reduce the saliva. We followed them up for 12 months. Four out of five patients had significant improvement of symptoms and quality of life, according to the criteria adopted for success, manifesting the desire to inject it again. In one of them, there was no reduction of saliva, but the patient presented the most severe affections with complete inability of oral cavity and oropharynx muscles. There were no systemic or local side effects. In three patients, improvement was prolonged up to the 4^th^ month and in one patient the action of Botox® was not detected anymore after the third month of application.

## DISCUSSION

Healthy subjects secrete from 1000 to 1500ml saliva within 24 hours³. Many neurological diseases progress with difficulties in the oral motor control. When the production of saliva exceeds the subjects’ skill to transport it from the mouth to the stomach, stasis, extraoral leak and aspiration may take place, in addition to concomitant difficulty of mastication and articulation. It is stigmatizing and the prevalence in neurological diseases is high. Sialorrhea occurs in about 50% of the patients with ALS and 20% of the victims need continuous saliva elimination ^2^, which has prevalence of about 70% in Parkinson disease ^4^, and between 10 and 80% in patients with cerebral palsy ^5^. The prevalence of sialorrhea in these affections is high, with impairment of social integration and difficulties to perform oral motor activities during feeding and speech, with repercussions in quality of life ^6^. We selected five patients with diagnosis of ALS for at least two years.

Among the treatment options for sialorrhea, anti-cholinergic drugs are the most widely used to reduce saliva, but there may be systemic side effects (urinary retention, loss of visual accommodation, headache, dry eyes), in addition to development of drug tolerance ^1^.

Another more recent option is the use of Botox® in PG and SG, despite the fact that botulinum toxin has a limited action, which is not recommended for treatment of chronic diseases, because of reapplications ^3^. In our opinion, it is a little invasive procedure, very discreet and without local or systemic adverse events, which means it is an excellent treatment approach.

The literature brings a wide range of aspects about the technique, dose, number of application points in the glands, selection of salivary glands to be treated, criteria of responses to treatment and side effects. Owing to the limited number of studies that objectively explain all those elements we would like to emphasize the importance of having a treatment protocol with standardized technique and the dose of applied botulinum toxin, plus the conduction of quantitative and qualitative studies of salivary secretion. The action of botulinum toxin in salivary glands inhibits the uptake of acetylcholine in the neuroglandular junction, however, differently from the neuromuscular action, other autonomic stimuli are responsible for saliva secretion ^7^.

In the review of the literature, the glands normally selected for Botox® application were isolated PG, or SG ^5^, the combination of both PG and SG at the same time ^7^, or still application in PG and if there is not the desired effect, reapplication made in PG and SG ^8^. Given that PG and SG are responsible for 80-90% of salivary secretion at rest, we decided to include the two groups at the same application, because the more the saliva were reduced, the better the results.

In the first reports, there was no description of the use of ultrasound to guide the application. More recent studies reported the difficulty to identify the SG with palpation, especially in children, and they reported that the accuracy of the method could be improved by having direct view of the needle under ultrasound guidance. The authors proposed the puncture to be guided by USG in PG and SG ^2^. Some authors advocated the administration of to PG in one single point 1, 6, two points^7^ or three points ^8^. Jongerius et al.. (2004) radiologically demonstrated that the best distribution of the substance in SG was the result of the application in two points.

The injected dose in PG ranged from 5U6 to 72U² of Botox®, and in the SG it ranged from 5U² to 50U^5^. We defined the dose of 20U in PG (distributed in two points) and 30U in SG (in one point).

We applied topical anesthesia for 30 minutes before the application. The authors that reported application under general anesthesia were Jongerius et al. (2004), because they carried out the study in children with the application of two points in the SG, with USG control, and probably accuracy was hindered because the children did not collaborate in the procedure.

Another controversial issue is the report of success that is described in all studies, regardless of doses and techniques. It may be justified by the subjectivity of the pre and post-application controls. In most studies, the success rate was given by subjective responses of the patient about the control of sialorrhea, in others by the measurement of dry cotton balls placed on the oral cavity before and after the application ^3^ and the most objective criterion that we observed was to analyze the results with the use of a quality of life questionnaire and salivary gland scintigraphy ^2^. Our criteria for subjective improvement was a four-question questionnaire answered by the patients that showed the reduction of extra-oral leak of saliva and social integration, leading to the inference about reduction of salivary secretion and quality of life improvement. Four of the five patients were success in their procedure. At the end of the follow up period, four patients manifested they wanted to reapply the injection, a situation that reflects the success of the procedure. The minimum time of action of Botox® in clinical responses was three months. The patient that did not show success in treatment had very advanced condition of the underlying disease, which was also reported by other authors ^2^. All authors have reported high success rates with Botox® in salivary glands of most of the studied patients.

We did not observe increase in the incidence of dental problems as a result of saliva reduction, despite the follow up period of only 12 months, but we emphasized the importance of careful dental hygiene and previous dental treatment before the application, because the saliva becomes thicker. Some authors reported that the reduction of salivary flow could increase the incidence of dental decays ^8^. Among the potential adverse events are xerostomia and worsening of dysphagia owing to diffusion of the drug to mastication muscles. We did not observe these adverse events in our patients.

## CONCLUSION

Application of 20U Botox® in parotid glands and 30U in submandibular glands with the technique advocated in the study was enough to reduce the saliva in patients and to improve the quality of life of ALS patients, comprising an alternative to the treatment of sialorrhea.
